# Dentists in the U. S. Army

**Published:** 1901-11-15

**Authors:** J. S. Marshall

**Affiliations:** San Francisco Cal.


### DENTISTS IN THE U. S. ARMY.

BY J. S. MARSHALL, M.D., D.D.S., SAN FRANCISCO CAL.

The following is a synopsis of the report made by Dr. J. S. Marshall, President of the Examining Board for Army Dental Surgeons, made to the National Dental Association at its meeting in Milwaukee, and from a paper read before the section on Stomatology of the

American Medical Association at St. Paul, Minn., last June.

The passage of the Army Reorganization Bill, by Congress, last winter, carried with it a scheme providing for the appointment of thirty dental surgeons to serve in the army, and also provided for their selection by a Board of Examiners, consisting of three members. Drs. Marshall, Oliver and Morgan in due time received the appointment to the Board.

The progress of organization has necessarily been slow, as the Board had no precedents to lead it, and it seemed important that as much care should be exercised as possible, so that no mistakes should be made and that the future of the movement might be secure.

The army dentist, although he holds his position on contract, belongs to the regular establishment and will serve the officers and privates of the army during certain hours each day, and is subject to the usual discipline and priveleges of a first lieutenant, with whom he is on equal rank. His pay is one hundred and fifty dollars per month and quarters when serving with the troops. All supplies and instruments being furnished. His hours for work are from 9 a.m. to 4 p.m. At other times he may engage in private practice.

The examinations for selection of suitable men have been placed on a high basis and no political or personal pull has had any place in making the selections.

The examinations consist of (*a*) physical condition; (*b*) written and oral questions upon the studies of the dental college course; (*c*) practical demonstrations in operative dentistry; (*d*) practical demonstrations in prosthetic dentistry.

(*a*) The physical examination is conducted by an army surgeon detailed for this purpose, and upon the same general lines as those in vogue for entrance into the other departments of the army. Perfect health and freedom from physical defect are necessary to pass this examination; but defective eyesight which can be corrected by appropriate glasses does not debar the candidate.

(*b*) Written and oral examinations are conducted upon the following-named subjects, and the candidate must attain a general average of seventy-five percent upon each of them; Anatomy, physiology, histology, chemistry, physics, metallurgy, dental anatomy and physiology, dental materia medica and therapeutics, dental pathology and bacteriology, orthodontia, oral surgery, operative dentistry, prosthetic dentistry.

(*c*) The practical examinations in operative dentistry consists of the following: 1. Examination and recording the condition of the mouth and teeth. 2. Preparation of cavities (*a*) by hand instruments; (*b*) by engine instruments. 3. Instrumentation and technique. 4. Preparation and manipulation of filling materials; gold, tin, amalgam, gutta-percha, oxyphosphate, cement. 5. Insertion and finishing of fillings. 6. Treatment and filling of root canals and preparation of root for pivot crown. 7. Manipulative technique in removal of calcareous deposits. 8. Application of rubber dam, metallic separators, matrices, etc. 9. Diagnosis, prognosis, and treatment of oral diseases. 10. Care of and sterilization of instruments and hands.

(*d*) The practical examination in prosthetic dentistry comprises: 1. Impressions, casts, bite and articulation (occlusion). 2. Construction of denture in vulcanite. 3. Construction of dies and counter-dies from impression to completion. 4. Construction of swaged plate, with metal and rubber attachment. 5. Construction of interdental splints. 6. Construction of Richmond crown.

Upon all clinical or practical demonstrations the candidate must attain a general average of eighty-five percent. The reason for this higher requirement in the practical branches is made evident by the statement that practical men are needed in the service, not theorists. But it may also be stated that practical ability alone would be of little value in military dental practice. The dentist to be successful in this new field of practice must be thoroughly informed upon all those subjects and theories which form the foundation of modern dental surgery. He will need to be self-reliant and capable of conducting any case that may come under his especial care, no matter how serious it may be, for he will many times be so located that he can not obtain the advise of a consultant in his specialty; while, upon the other hand, he may be, and that frequently, called in by the post surgeon as a consultant in cases which present oral or dental lesions; or which by reason of certain symptoms the surgeon is led to believe may be dependent upon some obscure dental or oral malady, and upon which he desires an expert opinion to assist him in an intelligent treatment of the case. The age limits are from twenty-four to forty years.

As rapidly as the candidates have passed the Examining Board they have been assigned to service in the Philippines and Cuba, for the reason that our troops located in tropical climates are in the greatest need of the services of the dentist. When these demands are satisfied in one location, the dentist will be assigned to another station, and thus moved about from place to place wherever his services are most urgently needed. The number of dentists is so few, thirty in all, and the army so large, something over seventy thousand, that there is likely to be an abundance of employment for all those who enter this service. The opportunity, however, for a large and varied scientific and practical experience along professional lines, offered by the establishment of the Army Dental Corps, will be very great, and ought to prove a tempting inducement to the really progressive young man.

The following regulations govern contract dental surgeons:

Candidates for appointment as dental surgeons must be not less than twenty-four nor more than forty years of age. They must be graduates of standard medical or dental colleges, trained in the several branches of dentistry, of good moral and professional character, and prior to appointment will be required to pass a satisfactory professional examination before a Board of Dental Surgeons convened for that purpose by the Secretary of War.

Contracts with dental surgeons will be made for three years, but may be annulled at any time by the commanding general of a military department, after official investigation, for conduct to the prejudice of good order and military discipline, or by the Surgeon-General when in his opinion a termination of the contract would be in the interest of the service.

Dental Surgeons are attached to the medical department and will be assigned to duty in accordance with the recommendations of the Surgeon-General of the army or the chief surgeon of a military department.

A dental surgeon when assigned to a station will apply to the post commander for a suitable operating-room. If no other room is available the surgeon of the post may assign him a room in the hospital.

Each dental surgeon will ordinarily be allowed one enlisted man as an assistant, who will be detailed from the acting hospital stewards or privates of the Hospital Corps, and whose duty it will be to assist the dentist in his operations, in caring for the instruments and other public property, in keeping the records, and in the performance of such other official work pertaining to this position as he may be directed by the proper authority to do. When a member of the Hospital Corps is detailed as dentist's assistant, he will receive commutation of rations at the rate of one dollar daily, and will be provided with a suitable room as quarters by the Quartermaster's Department, except while on duty at a post, when he will be attached to the Hospital Corps or other organization for rations and quarters. Necessary dental instruments and supplies will be purchased by medical supply officers under instructions from the Surgeon-General and in accordance with a supply table to be approved by the Secretary of War.

Dental surgeons will be held strictly responsible for all instruments and supplies issued to them, and will be governed by army regulations and orders now in force, or hereafter to be issued, with reference to accountability for government property.

In accordance with the act of Congress authorizing their employment, dental surgeons will "serve the officers and enlisted men of the regular and volunteer army." The families of officers and civilian employees attached to the army are not entitled to their services. In this connection acting assistant surgeons are to be regarded as commissioned officers.

Dental surgeons will operate between the hours of nine a.m. and four p.m. only upon those officers and enlisted men who are entitled to their services. They may operate upon others not entitled to free service before and after these hours when their services are not required by those entitled to them, but material issued to them by the government will only be used in operations upon officers and enlisted men of the army.

Dental surgeons will not perform any operation upon officers or enlisted men of the army or prescribe medicines for them other than those necessary for the treatment of the teeth and gums. This prohibition does not apply to cases of emergency where no medical officer is within reach, and where a dental surgeon is able to render the neccessary surgical assistance to meet the immediate emergency.

Emergency work, whether for officers or enlisted men, should always have precedence. Plate work or restoration of teeth by any method will only be done for those who have lost teeth in the service and in the line of duty. For plate work or filling teeth only the cheaper materials will be supplied, but gold may be used, if the operating dentist sees fit to use it, at the expense of the individual operated upon.

Enlisted men requiring the services of the dental surgeon will, at an hour prescribed by the commanding officer, be conducted to the designated place under a non-commissioned officer, who will take with him and hand to the dentist a list of those reporting for treatment. This list will be entered in a daybook ruled in columns for surname, given name, rank, company, regiment, etc.; all headings to be the same as those borne on his monthly report.

All cases requiring treatment involving future appointment will be noted, and the others will be marked according to the circumstances, as "treatment unnecessary," "further treatment unnecessary," "should be sent to surgeon,'' etc. When future treatment is necessary the dentist will forward a card as follows:

........................19..

THE ADJUTANT:

..........................................

SIR—I have the honor to ask that ......................
be directed to report to me from ...m. to ...m. on........
instant for treatment. Very respectfully,

..........................

*Dental Surgeon.*

Dental surgeons will submit a monthly report in duplicate (on prescribed blanks) of all official work done by them, giving all required data in every case in which professional services have been rendered. This report will be an exact copy of the register kept for the period. One copy will be sent on the last day of the month to the Surgeon-General and one to the chief surgeon of the department in which the dental surgeon is serving.

The field outfit includes a portable dental chair and a dental engine, packed in separate cases; burs, mandrels, stones, disks, etc., excavators, chisels, scalers, plastic-pluggers, gold-pluggers, rubber-dam clamps, clamp forceps, dam punch, extracting forceps, elevators, steam sterilizer, etc.; in fact, all of the instruments and adjuncts that are really necessary to perform any operation upon the teeth, except for crowns and bridges. Each outfit contains medicines and supplies of filling-material sufficient for three months' service. The smaller instruments and the supplies are packed in two strong cases, arranged with trays and receptacles to hold the instruments and supplies in place. The whole outfit when cased and crated weighs about four hundred and fifty pounds. To protect the cases from rain and dampness they are enclosed in canvas covers. The general hospitals, and such other posts as may be designated by the Surgeon-General, will be furnished with an additional outfit, consisting of a regular office operating chair, Allen bracket, cuspidor, instrument case, extra extracting forceps, and a full laboratory outfit for constructing vulcanite plates, swaged metal plates, crowns and bridges.

Up to the time this report was made eighty-six dentists had been invited by the Surgeon-General to present themselves for examination. Of this number sixteen declined to appear. Out of the seventy examined nineteen were found qualified and recommended for appointment. Eight of the seventy were found physically disquallified, three were rejected of a full examination on all requirements, seven failed on the theoretical examination, thirty-three withdrew without completing the full examination. The average age of the nineteen appointed is twenty-seven and one-half years. One is a graduate of medicine. In accordance with the clause in the law which made it compulsory to appoint such men as were already in service, without examination, five were appointed in this way, thus making twenty-seven appointments including the three members of the Board. This leaves three vacancies to be filled.

The appointments are from sixteen states; one from Alabama, one from California, two from the District of Columbia, one from Iowa, one from Maryland, one from Massachusetts, two from Missouri, one from Montana, one from Nebraska, one from New Jersey, one from New York, one from Ohio, two from Pennsylvania, one from Washington, one from West Virginia, and one from Wisconsin.

They have been distributed to the following stations: J. S. Marshall, Ft. Presidio, San Francisco, Cal.; R.

T. Oliver, Manila, P. I.; R. W. Morgan, Havana, Cuba; R. P. Updike, Ft. Leavenworth, Kas.; E. P. Liquor, Ft. Riley, Kas.; W. H. Ware, P. I.; H. C. Rietz, P. I.; O. M. Sorber, Ft. Sam Huston, Tex.; W. C. Fisher, Ft. Sheridan, Ills.; J. W. Hess, West Point, N. Y.; R. W. Waddell, P. I.; S. D. Broak, P. I.; F. F. Wing, P. I.; F. P. Stone, P. I.; F. H. Wolvin, P. I.; G. M. Decker, Cuba; W. H. Chambers, Ft. Monroe, Va.; G. L. Mason, P. I.; C. E. Lauderdale, P. I.; J. C. Winnery, P. I.; C J. Long, P. I.; H. G. Voorheis, San Juan, Porto Rico; E. J. Craig, P. I.; S. W. Hussey, P. I.; C. A. Petre, P. I.; D. E. Foster, P. I.; A. Carpenter, P. I.

While the examinations were directed chiefly to determine the technical skill of candidates, the theoretical examinations was based upon a high standard. This part of the examination showed that theoretical subjects are not as thoroughly taught as they should be.

### DISCUSSION.

Dr. B. H. Smith, of Baltimore: It is very appropriate that this body should have a report of the proceedings of this Board of Examiners, as no other organization can be more interested in this work or has had so much to do with bringing this result to pass, and we should be grateful that the United States government concedes this point. I can vouch for the thoroughness of the work of this Board as I have seen a number of its battle scarred and defeated victims retreating to a more quiet sphere of usefulness. I would suggest that we send a petition to the Surgeon-General asking that hereafter candidates for army positions must have the endorsement of their state, or district societies before they are permitted to come up for examination.

Dr. William Donnelly, of Washington, D. C.: This subject has largely absorbed my attention for many years, and because of my connection with this work I feel that I ought to have the privilege of speaking on this subject. I do not care to criticise the Board or its actions, although I deprecate the fact that the appointments were made wholly on a political basis. The Board ought to be made up of men of the strictest integrity, as it has the power of rejecting a candidate even though he may have satisfactorily passed the examinations. I hope the Army Dental Surgeons will be discreet enough to avoid collisions with the medical surgeons, such as might occur from encroaching on each others provinces. This might result disastrously to the future of the service by creating a prejudice which we can not afford to have to work against at this time. The dental surgeon should not even offer to do work which we hope may in the very near future be delegated to him, but which is at present ordinarily done by the medical surgeon. Unless some unfortunate accident occurs I believe the Army Dental Surgeon has come to stay in spite of the fact that he is a non-commissioned officer.

Dr. Oliver: Speaking as a member of the Examining Board I want to say that the meetings of the Board have been carried on without the least trouble or discord. We have tried to make our examinations as fair as possible and no candidate has had the slightest advantage because of any political pull he may have had. Every man stood or fell on his merit as shown by the examinations. It is true that we advised several candidates to withdraw before they had completed the examinations, for the reason that it was evident they must fail, and we did not consider it right to keep them on expense unnecessarily. The standard was made high purposely, with the idea in mind of securing the best possible grade of men for army dental surgeons.

Dr. J. Taft, of Cincinnati, moved that a vote of thanks be extended the Board for so faithfully and conscientiously performing the work entrusted to it. The motion was unanimously carried by a rising vote.

